# Sesamol intervention ameliorates obesity-associated metabolic disorders by regulating hepatic lipid metabolism in high-fat diet-induced obese mice

**DOI:** 10.29219/fnr.v63.3637

**Published:** 2019-10-23

**Authors:** Hong Qin, Haiyan Xu, Liang Yu, Lina Yang, Cui Lin, Jihua Chen

**Affiliations:** 1 Department of Nutrition Science and Food Hygiene, Xiangya School of Public Health, Central South University, Changsha, China;; 2 Department of Research and Development Office, Hunan First Normal University, Changsha, China

**Keywords:** sesamol, obesity, liver, lipid metabolism

## Abstract

**Background:**

Obesity has currently become a serious social problem to be solved. Sesamol, a natural bioactive substance extracted from sesame oil, has shown multiple physiological functions, and it might have an effect on the treatment of obesity.

**Objective:**

This study was conducted to investigate the therapeutic effect and potential mechanisms of sesamol on the treatment of obesity and metabolic disorders in high-fat diet (HFD)-induced obese mice.

**Methods:**

C57BL/6J male mice were fed HFD for 8 weeks to induce obesity, followed by supplementation with sesamol (100 mg/kg body weight [b.w.]/day [d] by gavage) for another 4 weeks. Hematoxylin and eosin staining was used to observe lipid accumulation in adipose tissues and liver. Chemistry reagent kits were used to measure serum lipids, hepatic lipids, serum alanine aminotransferase (ALT), and aspartate aminotransferase (AST) levels. ELISA kits were used to determine the serum insulin and free fatty acid (FFA) levels. Western blotting was used to detect the protein levels involved in lipid metabolism in the liver.

**Results:**

Sesamol significantly reduced the body weight gain of obese mice and suppressed lipid accumulation in adipose tissue and liver. Sesamol also improved serum and hepatic lipid profiles, and increased insulin sensitivity. In the sesamol-treated group, the levels of serum ALT and AST decreased significantly. Furthermore, after sesamol treatment, the hepatic sterol regulatory element binding protein-1 (SREBP-1c) decreased, while the phosphorylated hormone sensitive lipase (p-HSL), the carnitine palmitoyltransferase 1α (CPT1α), and the peroxisome proliferator-activated receptor coactivator-1α (PGC1α) increased, which were responsible for the fatty acid synthesis, lipolysis, and fatty acid β-oxidation, respectively.

**Conclusions:**

Sesamol had a positive effect on anti-obesity and ameliorated the metabolic disorders of obese mice. The possible mechanism of sesamol might be the regulation of lipid metabolism in the liver.

## Popular scientific summary

- Sesamol significantly lowered body weight and ameliorated dyslipidemia, insulin resistance, and hepatic steatosis in high-fat diet-induced obese mice; and it might be due to the regulation of hepatic lipid metabolism by sesamol.
- The liver is known to involve in lipid metabolism, including lipid uptake, lipid synthesis, and lipid catabolism. Sesamol reduced hepatic lipogenesis and increased lipolysis and fatty acid β-oxidation, which in turn reduce lipid accumulation in the body to alleviate obesity and obesity-associated metabolic disorders.

Obesity has become a serious public health concern that threatens human health worldwide (1, 2). The incidence of obesity-associated metabolic diseases, such as hypertension, type 2 diabetes, and hepatic steatosis, is rapidly increasing (3–5). Therefore, effective prevention and treatment strategies are needed to halt the development of obesity.

For the treatment of obesity, promoting lipid metabolism is an attractive way (6–8). It is well-known that liver is one of the main organs responsible for lipid metabolism, including lipid uptake, lipid synthesis, and lipid catabolism (9–12). Previous studies have demonstrated that regulating the factors responsible for lipid metabolism in liver could protect the body against obesity (13–15).

Sesamol, a natural bioactive substance extracted from sesame oil (16), has been reported to exhibit multiple biological effects (17–20). Recently, it is reported that the administration of sesamol significantly reduced the body weight in HFD-induced obese mice and decreased lipid droplets accumulation in 3T3-L1 adipocytes (21, 22). These findings suggested that sesamol had a potential anti-obesity effect. However, the detailed mechanism remains unclear, especially the effect of sesamol on hepatic lipid metabolism has not been clearly defined. In this study, we investigated the effects of sesamol on the aspect of hepatic lipid metabolism in HFD-induced obese mice in order to elucidate the underlying mechanism of sesamol in the treatment of obesity and obesity-associated metabolic disorders.

## Materials and methods

### Animals and diet

Male C57BL/6J mice (4–6 weeks old) were purchased from Central South University (Hunan, Changsha, China). The mice were housed in the standard animal room (humidity: 40%–70%, temperature: 22 ± 2°C, light and dark cycle: 12 h/12 h, free feeding and drinking water). After acclimatization, one group of the mice were fed with the normal-fat diet (NFD, 10 kcal% fat; rodent diet D12450B, Research Diets, New Brunswick, NJ, USA), and all the other mice were fed with a HFD (60 kcal% fat; rodent diet D12492, Research Diets, New Brunswick, NJ, USA). After 8 weeks, mice weighing 20% more than the average weight of mice in the NFD group were considered obese mice, and then, the obese mice were divided into two groups: HFD group (fed a HFD) and HFD+sesamol group (fed a HFD and administered with sesamol by gavage). All three groups with eight mice in each were fed for another 4 weeks. Sesamol (Sigma-Aldrich, St. Louis, MO) was dissolved in a vehicle (0.5% carboxylmethyl cellulose) and administered at a dose of 100 mg/kg b.w./d by gavage, and mice in the NFD and HFD groups were given the equal volume of vehicle by gavage. The mice were allowed free access to water and food. Everyday food-intake and every-week body weight of the mice were recorded. The project was approved by the Animal Care and Use Committee of Central South University (IACUC Approval Number: 2019sydw0025), and all experiments were performed in accordance with the committee’s guidelines.

### Intraperitoneal glucose tolerance test (IPGTT), fasting blood glucose (FBG), serum insulin, and the insulin resistance index (HOMA-IR)

The IPGTT was performed in the 12th week, and mice were fasted for 12 h before the experiment. The FBG was measured with tail vein blood using a glucose analyzer (Contour TS, Bayer, Germany). Then, the mice were intraperitoneally injected with 2 g/kg body weight glucose, and then, the blood glucose levels were measured with tail vein blood at 15, 30, 60, and 90 min. ELISA assay was used to measure the serum insulin concentration (cusabio, WuHan, China). The homeostasis model assessment of HOMA-IR was calculated as follows: fasting insulin concentration (mU/L) × fasting glucose concentration (mmol/L)/22.5.

### Serum and tissue collection

After 12 weeks, the mice were anesthetized with ether. Blood samples were taken from the femoral artery and placed at room temperature for 30 min and centrifuged at 2,500 × rpm for 10 min, and separated serum samples were stored at −80°C. Then, the mice were killed by cervical dislocation. The livers and adipose tissues (epididymal, perirenal, and inguinal white adipose tissue [WAT]) were dissected, rinsed, and weighed. Then, the livers and adipose tissues were divided into two parts: one was quickly fixed in 4% formalin solution for histological analysis, and the other was quickly frozen in liquid nitrogen and immediately stored at −80°C until use.

### Histological analysis

Fixed liver and adipose tissue were removed from formalin solution and embedded in paraffin wax. Paraffin-embedded sections (5 μm) were stained with hematoxylin and eosin (HE). Then, the sections were analyzed by an optical microscope (EVOSTM Auto2, Thermo Fisher Scientific, WA, USA). Adipocyte size was determined by measuring the area of 100 adipocytes in stained sections and analyzed with Image J software.

### Serum biochemical analyses

Serum levels of total cholesterol (TC), low-density lipoprotein cholesterol (LDL-C), high-density lipoprotein cholesterol (HDL-C), serum-free fatty acid (FFA), ALT, and AST were measured using chemistry reagent kits (Nanjing Jiancheng, Nanjing, China).

### Hepatic lipid concentration

The liver samples were homogenized with normal saline (1:9, w/v) and centrifuged at 2,500 rpm for 10 min. Hepatic triglycerides (TG), TC, and LDL-C levels were assayed using the same kits used for serum analysis.

### Western blotting analysis

Liver tissues were harvested in a cold RIPA buffer containing proteinase inhibitor (Ding Guo Changsheng Biotechnology Co., Ltd., Beijing, China). All protein extracts were then centrifuged at 12,000 rpm at 4°C for 15 min. The protein concentration of supernatants was measured with BCA Protein Assay Kit (Beyotime Biotechnology, Shanghai, China).

The protein was separated by sodium dodecylsulfate polyacrylamide gel electrophoresis (SDS-PAGE) and transferred to polyvinylidene fluoride (PVDF) membranes (Bioleader, Maine, USA). After blocked in 5% skim milk in Tris-buffered saline containing 0.1% Tween-20 (TBST) for 1 h at room temperature, the membranes were incubated overnight at 4°C with primary antibodies and secondary antibody for 1 h at room temperature. With an ECL method, protein bands were detected using the chemiluminescence imager (Tanon-5500, Tanon Science & Technology Co., Ltd., Shanghai, China). The primary antibodies used in this study were SREBP-1c (A15586), CPT1α (A5307), PKA-C (A7715), and β-actin (AC026), which were purchased from Abclonal (Wuhan, China); and HSL (AF6403) and Phospho-HSL (p-HSL, AF8026) were purchased from Affinite (OH., USA). The secondary antibody was HRP Goat Anti-Rabbit IgG (AS014) purchased from Abclonal.

### Statistical analysis

Data are expressed as the mean ± standard deviations (SD). Statistical analysis was performed using SPSS version 18.0 (SPSS Inc., Chicago, IL, USA). One-way analysis of variance (ANOVA) was used for comparisons among group. Significant differences between the mean values were assessed using least-significant difference *t*-test (LSD-*t*). Results were considered statistically significant at *P* < 0.05.

## Results

### Effect of sesamol on body weight in obese mice

**Fig. 1:**
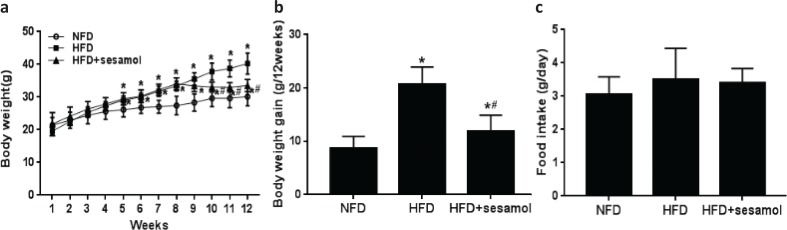
Effect of sesamol on the body weight in HFD-induced obese mice. (a) Body weight, (b) body weight gain, and (c) food intake based on food consumption per day per mouse. Data are shown as mean ± SD (*n* = 8). * *P* < 0.05 compared with the NFD group; # *P* < 0.05 compared with the HFD group.

After 8 weeks, the weights of the mice fed with HFD were significantly higher than the mice fed with NFD, and the 4-week treatment with sesamol markedly decreased the weights of the mice (Fig. 1a), and the body weight gain of HFD+sesamol group was significantly lower than that of the HFD group (Fig. 1b). In addition, there was no significant difference in the food intake among the three groups (Fig. 1c).

### Effects of sesamol on adipose tissue in obese mice

**Fig. 2:**
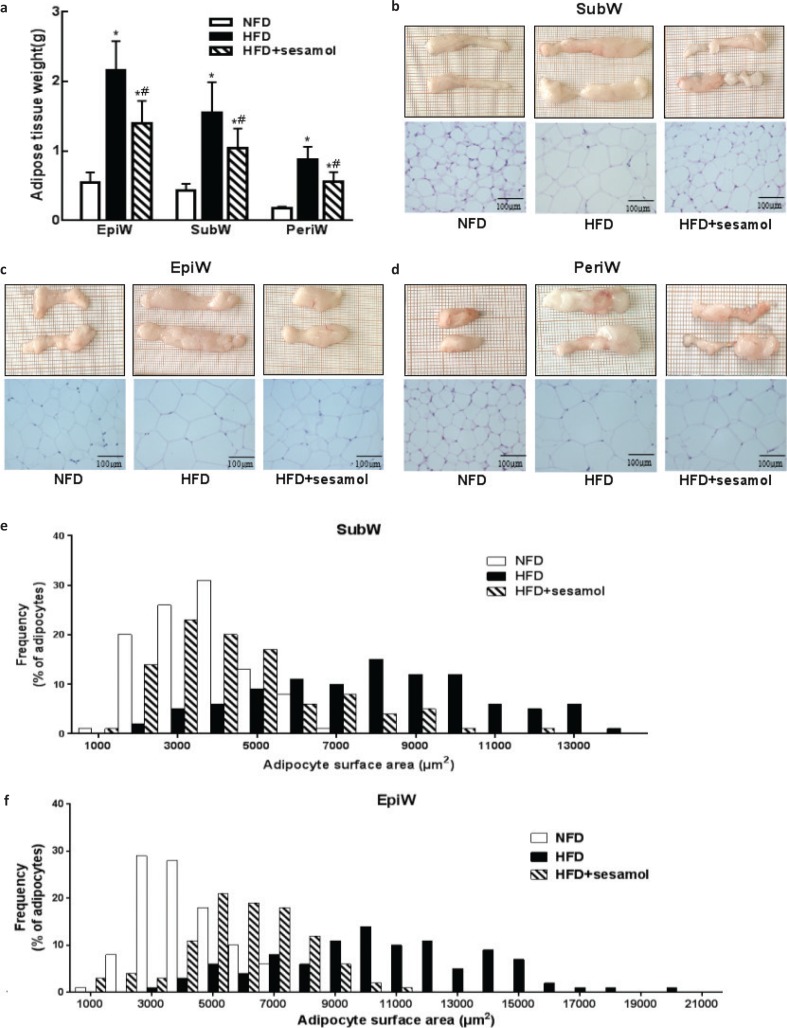
Effects of sesamol on white adipose tissue (WAT) weights and histological analysis in HFD-induced obese mice. (a) The weights of WATs. (b) SubW, (c) EpiW, and (d) PeriW morphology and H&E staining (400×), scale bar = 100 μm. (e) The size distribution of adipocytes in SubW. (f) The size distribution of adipocytes in EpiW. SubW: inguinal WAT; EpiW: epididymal WAT; PeriW: perirenal WAT. Data are shown as mean ± SD (*n* = 8). * *P* < 0.05 compared with the NFD group; # *P* < 0.05 compared with the HFD group.

The epididymal, perirenal, and inguinal WAT weights were significantly reduced by sesamol compared with the HFD group (Fig. 2a). Adipose tissues were smaller in terms of appearance after sesamol treatment, and sections showed that the sizes of adipocytes were also smaller in the HFD+sesamol group than that in the HFD group (Fig. 2b–d). Furthermore, the cell size distributions of representative WATs were quantified. We found that there was a shift from larger cell area to smaller cell area in adipose tissues in sesamol-treated mice (Fig. 2e, f). These results indicated that sesamol might reduce lipid storage in adipose tissues in HFD-induced obese mice.

### Effect of sesamol on IPGTT, FBG, serum insulin, and the HOMA-IR in obese mice

**Fig. 3:**
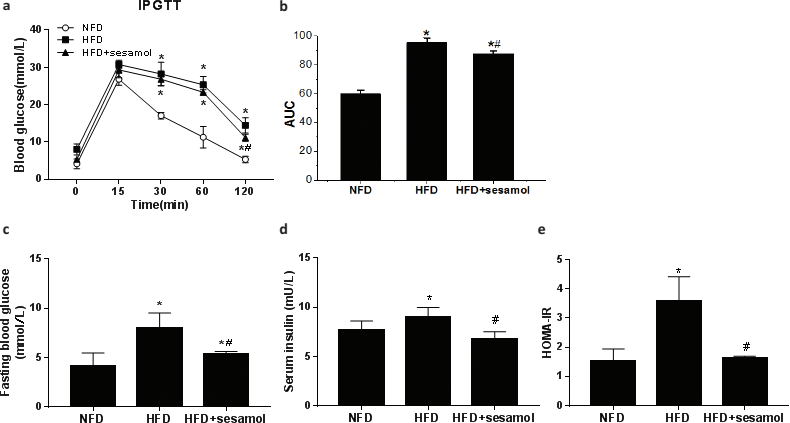
Effect of sesamol on glucose tolerance and insulin resistance in HFD-induced obese mice. (a) The blood glucose concentration at the indicated times (0, 15, 30, 60, and 120 min) after intraperitoneal injection of glucose. (b) Area under the curve (AUC) of blood glucose levels after the IPGTT. (c) Blood glucose levels after 12-h fasting. (d) Serum insulin levels after 12-h fasting. (e) HOMA-IR calculated by fasting blood glucose and insulin levels. Data are shown as mean ± SD (*n* = 3). * *P* < 0.05 compared with the NFD group; # *P* < 0.05 compared with the HFD group.

Glucose disposal was delayed in IPGTT, and FBG was significantly increased in the HFD group compared with the NFD group, which indicated that HFD feeding induced glucose intolerance in mice, and sesamol improved glucose tolerance by promoting glucose disposal and decreasing FBG (Fig. 3a–c). The serum insulin level and HOMA-IR were significantly higher in the HFD group than that in the NFD group, whereas sesamol markedly reduced serum insulin levels and HOMA-IR (Fig. 3d, e), which indicated an improvement of insulin sensitivity after sesamol treatment.

### Effects of sesamol on serum lipid profile in obese mice

**Fig. 4:**
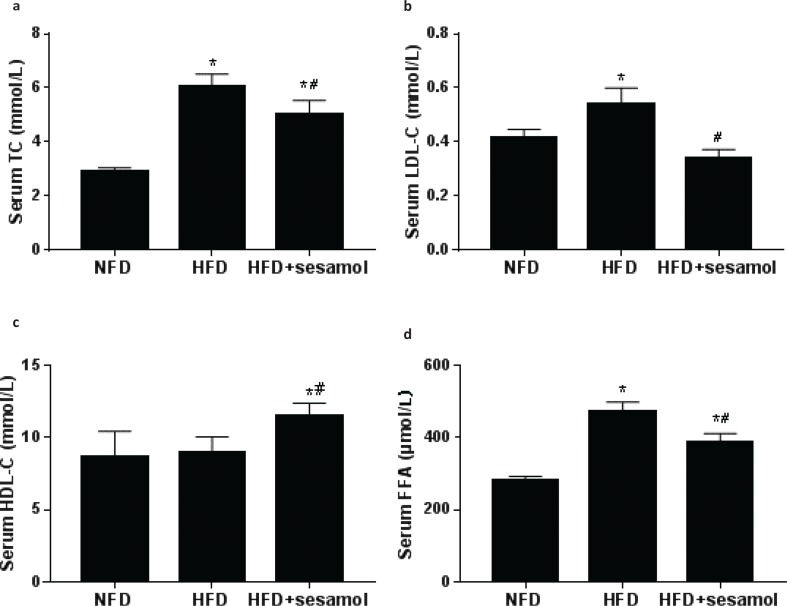
Effects of sesamol on serum lipid levels in HFD-induced obese mice. (a) Serum TC, (b) serum LDL-C, (c) serum HDL-C, and (d) serum FFA. Data are shown as mean ± SD (*n* = 3). * *P* < 0.05 compared with the NFD group; # *P* < 0.05 compared with the HFD group.

The levels of serum TC and LDL-C were dramatically lower in the HFD+sesamol group compared with the HFD group (Fig. 4a, b). The level of HDL-C was significantly higher in the HFD+sesamol group (Fig. 4c). Additionally, the level of serum FFA was significantly decreased by sesamol compared with the HFD group (Fig. 4d). These data suggested that HFD-induced dyslipidemia was significantly improved by sesamol.

### Effects of sesamol on liver function and lipid profile in obese mice

**Fig. 5:**
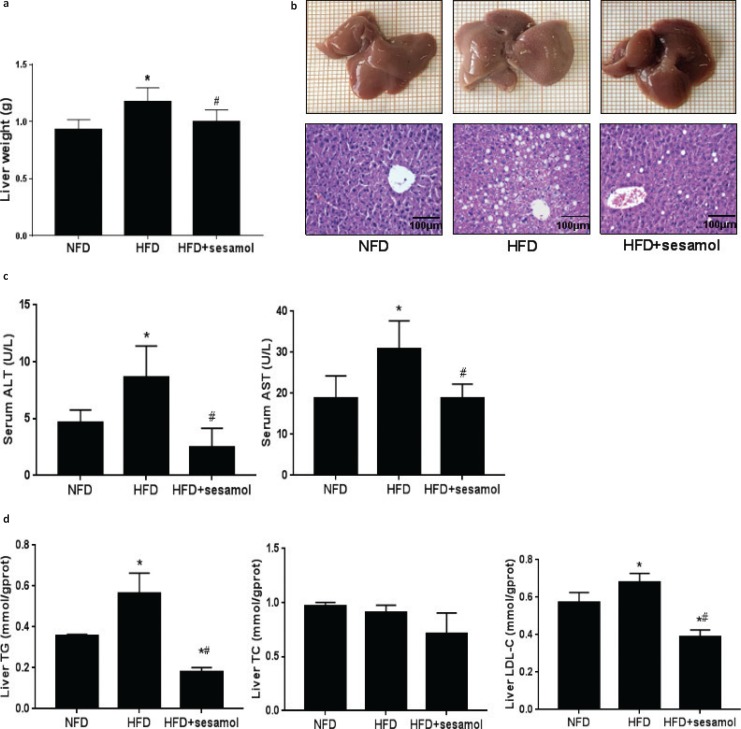
Effects of sesamol on liver histology, liver weight, liver function, and liver lipid profiles in HFD-induced obese mice. (a) Liver weight (*n* = 8). (b) Liver morphology and sections of H&E staining (400×), scale bar = 100 μm. (c) Serum ALT and AST. (d) Liver TG, TC, and LDL-C. Data are shown as mean ± SD (*n* = 3). * P < 0.05 compared with the NFD group; # P < 0.05 compared with the HFD group.

The liver weight was obviously decreased in the HFD+sesamol group compared with the HFD group (Fig. 5a). In terms of liver morphology, the livers were pale and larger in the HFD group, which was improved after sesamol treatment, and liver sections showed that hepatic fat vacuoles were also much fewer in the HFD+sesamol group than the HFD group (Fig. 5b). Serum ALT and AST levels were markedly reduced in the HFD+sesamol group (Fig. 5c). Furthermore, the levels of hepatic TG and LDL-C were also significantly lower after sesamol treatment (Fig. 5d). Thus, we concluded that sesamol could decrease lipid accumulation in the liver and improve liver function.

### Effects of sesamol on hepatic lipid metabolism regulators in obese mice

**Fig. 6:**
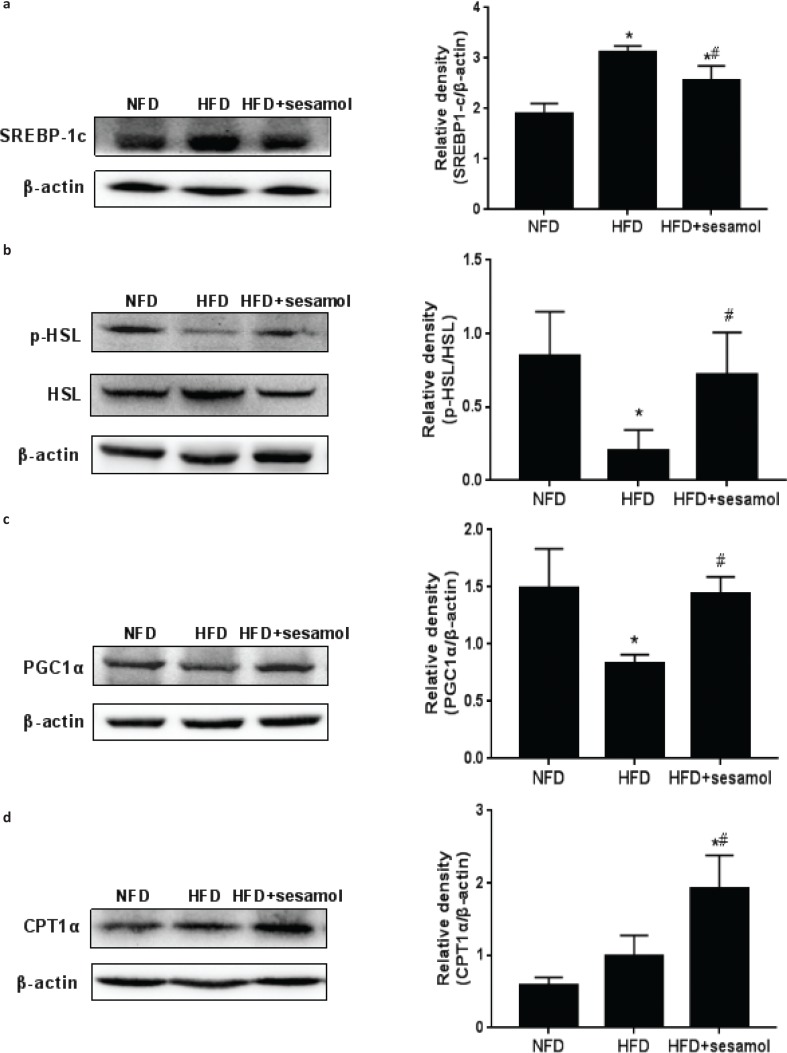
Effects of sesamol on protein expression levels related to lipid metabolism in HFD-induced obese mice. Protein expression levels and density analysis of (a) SREBP-1c, (b) p-HSL and HSL, (c) PGC1α, and (d) CPT1α. Data are shown as mean ± SD (*n* = 3). * *P* < 0.05 compared with the NFD group; # *P* < 0.05 compared with the HFD group.

Since liver plays a critical role in metabolism, several representative hepatic lipid metabolism regulators were detected to explore the anti-obesity mechanism of sesamol. The expression of SREBP-1c was significantly reduced in the HFD+sesamol group compared with the HFD group (Fig. 6a), which indicated a decrease in fat synthesis. The expression of p-HSL was markedly increased by sesamol (Fig. 6b); in addition, PGC1α and CPT1α expression levels were higher in the HFD+sesamol group than that in the HFD group (Fig. 6c, d), which demonstrated the enhancement of lipolysis and fatty acid oxidation. Collectively, these data suggested that sesamol might be involved in hepatic lipid metabolism.

## Discussion

Increasing attentions have been paid to phytochemicals that serve as alternative strategies for obesity treatment (23, 24). In this study, we investigated the effects of sesamol on HFD-induced obese mice and explored the underlying mechanisms. For the first time, we demonstrated that sesamol could reduce body weight gain and improve obesity-associated metabolic disorders, which were dependent on the regulation of lipid metabolism in the liver, and the detailed mechanism was that sesamol down-regulated SREBP-1c and up-regulated p-HSL, PGC1α, and CPT1α in the liver.

Obesity is characterized by increased lipid storage in an expanded adipose tissue mass (25). In this study, we found that sesamol markedly reduced the body weight gain of HFD-induced obese mice without affecting the food intake of mice, and it also reduced weights of epididymal, perirenal, and inguinal WAT and diminished adipocyte size. These results directly proved that sesamol had an anti-obesity effect. Notably, it has been reported that obesity is closely related to the development of metabolic disorders, including dyslipidemia, insulin resistance, and hepatic steatosis (26). Our results showed that sesamol decreased the levels of serum TC and LDL-C and increased serum HDL-C level to alleviate dyslipidemia in obese mice. Additionally, sesamol promoted glucose disposal and decreased FBG level, which suggested that sesamol improved the glucose tolerance of obese mice. Sesamol also decreased serum insulin level and HOMA-IR, which indicated that sesamol could enhance insulin sensitivity. These results illustrated that sesamol had an effective anti-obesity effect and ameliorated obesity-associated metabolic disorders.

The liver plays a key role in lipid homeostasis (27); however, excessive fat tends to accumulate in the liver in a state of obesity, which impairs liver function and further exacerbates obesity-associated metabolic disorders (28, 29). Therefore, we sought to determine whether sesamol could reduce fat accumulation in the liver to treat obesity and obesity-associated metabolic disorders. The results showed that livers weight and fat vacuoles in liver sections were obviously reduced, which indicated that sesamol alleviated obesity-associated hepatic steatosis. We also found that serum ALT and AST were decreased by sesamol, and liver TG and LDL-C levels were significantly reduced by sesamol, which suggested that the recovery of liver function benefited from the reduction of hepatic lipid accumulation in the presence of sesamol.

Since the liver is involved in fat synthesis, lipolysis, and oxidation (30, 31), we next investigated whether sesamol could directly regulate hepatic lipid metabolism regulators to reduce lipid accumulation in the whole body, which further clarified the mechanism of sesamol in anti-obesity. It is well-known that lipogenesis is transcriptionally regulated by SREBP-1c, which is a critical transcription factor and stimulates the expression of lipogenic enzymes involved in lipid synthesis in the liver (32). In this study, we found higher expression of SREBP-1c in the liver of obese mice, and sesamol markedly down-regulated SREBP-1c expression level. Given previous studies have demonstrated that decreasing lipid synthesis in the liver ameliorated obesity and hepatic steatosis (33, 34), we believed that sesamol might reduce fat accumulation in the whole body by reducing fat synthesis in the liver of obese mice.

Except for a decrease in fat synthesis, the reduction of fat accumulation in the liver might also be contributed by an increase in lipolysis. Thus, we detected the expression of HSL in the liver. It is well-known that HSL, a rate-limiting enzyme for TG decomposition, needs to be phosphorylated for lipolysis (35, 36). Furthermore, it has been proved that HSL-mediated lipolysis can ameliorate hepatic steatosis (37). In our study, sesamol obviously increased p-HSL expression level in the liver, which proved that sesamol could indeed promote lipolysis in the liver.

Furthermore, lipolysis releases FFA that is partly eliminated by β-oxidation, and the rest goes into the blood circulation (38). However, an increase in lipolysis releases excessive FFA that leads to lipotoxicity by impairing cellular signaling and function, which results in decreasing insulin sensitivity (39, 40). In our study, although lipolysis in the liver was enhanced by sesamol, we observed a decrease in serum FFA and improvement in insulin sensitivity, so we hypothesized that sesamol might increase fatty acid β-oxidation in the liver. As is known, fatty acid β-oxidation occurs in mitochondria (41), PGC1α is related to mitochondrial biosynthesis (42, 43), and CPT1α is a key enzyme for fatty acid β-oxidation in the mitochondria, which catalyzes the transport of fatty acid to mitochondrial matrix for oxidation (44, 45). Therefore, we detected the expressions of PGC1α and CPT1α in the liver and found that sesamol up-regulated PGC1α and CPT1α expression levels. These results confirmed that sesamol indeed enhanced fatty acid β-oxidation in the liver.

Our findings demonstrated that sesamol decreased the expression of SREBP-1c and increased the expressions of p-HSL, PGC1α, and CPT1α, which promoted lipid metabolism in the liver to ameliorate obesity and obesity-associated metabolic disorders. Notably, obesity is simultaneously caused by internal (genetic) and external factors (dietary or lifestyle) in some cases, and it was reported that ob/ob mice, a hereditary obesity model caused by genetic mutation, had hepatic steatosis related to the increase of SREBP-1c level and the decrease of PGC1α and CPT1α levels in the liver (46–48). Considering the positive effects of sesamol on these proteins in the liver of HFD-induced obese mice, we suspect that these effects could be expected in ob/ob mice. In future research, we would further determine the therapeutic effect of sesamol on obesity in ob/ob mice. Moreover, in this study, we chose mice to be the experimental subject in line with numerous studies about lipid metabolism in obesity state (5, 49); however, the results in animal experiments do not necessarily reflect the situation in human beings. Therefore, incorporating in vivo and in vitro experiments that we would like to perform using human hepatic cell lines, such as HepG2 and L02 cells, will provide a basis for clinical experiments of sesamol in order to develop sesamol as a drug for human obesity treatment in the future.

## Conclusions

Collectively, this study revealed that sesamol could ameliorate obesity and obesity-associated metabolic disorders through regulating hepatic lipid metabolism, including decreasing lipogenesis and increasing lipolysis and fatty acid β-oxidation in the liver of obese mice. Our results suggested that sesamol might serve as a versatile drug to treat obesity.

## References

[1] Lee YS, Kim SH, Yuk HJ, Lee GJ, Kim DS Tetragonia tetragonoides (Pall.) Kuntze (New Zealand Spinach) prevents obesity and hyperuricemia in high-fat diet-induced obese mice. Nutrients 2018; 10: 1087. doi: 10.3390/nu10081087. PMC6116159 30110943

[2] Smith KB, Smith MS Obesity statistics. Primary Care 2016; 43: 121–35. doi: 10.1016/j.pop.2015.10.001. 26896205

[3] Langhi C, Arias N, Rajamoorthi A, Basta J, Lee RG, Baldan A Therapeutic silencing of fat-specific protein 27 improves glycemic control in mouse models of obesity and insulin resistance. J Lipid Res 2017; 58: 81–91. doi: 10.1194/jlr.M069799. 27884961 PMC5234712

[4] Liang YC, Yang MT, Lin CJ, Chang CL, Yang WC Bidens pilosa and its active compound inhibit adipogenesis and lipid accumulation via down-modulation of the C/EBP and PPARγ pathways. Sci Rep 2016; 6: 24285. doi: 10.1038/srep24285. 27063434 PMC4827119

[5] Ma X, Xu L, Alberobello AT, Gavrilova O, Bagattin A, Skarulis M, et al. Celastrol protects against obesity and metabolic dysfunction through activation of a HSF1-PGC1α transcriptional axis. Cell Metab 2015; 22: 695–708. doi: 10.1016/j.cmet.2015.08.005. 26344102

[6] Song NJ, Yun UJ, Yang S, Wu C, Seo CR, Gwon AR, et al. Notch1 deficiency decreases hepatic lipid accumulation by induction of fatty acid oxidation. Sci Rep 2016; 6: 19377. doi: 10.1038/srep19377. 26786165 PMC4726366

[7] Wang L, Zhang B, Huang F, Liu B, Xie Y Curcumin inhibits lipolysis via suppression of ER stress in adipose tissue and prevents hepatic insulin resistance. J Lipid Res 2016; 57: 1243–55. doi: 10.1194/jlr.M067397. 27220352 PMC4918853

[8] Shiomi Y, Yamauchi T, Iwabu M, Okada-Iwabu M, Nakayama R, Orikawa Y, et al. A novel Peroxisome Proliferator-Activated Receptor (PPAR)α agonist and PPARγ antagonist, Z-551, ameliorates high-fat diet-induced obesity and metabolic disorders in mice. J Biol Chem 2015; 290: 14567–81. doi: 10.1074/jbc.M114.622191. 25907553 PMC4505524

[9] Yang MY, Chan KC, Lee YJ, Chang XZ, Wu CH, Wang CJ Sechium edule shoot extracts and active components improve obesity and a fatty liver that involved reducing hepatic lipogenesis and adipogenesis in high-fat-diet-fed rats. J Agric Food Chem 2015; 63: 4587–96. doi: 10.1021/acs.jafc.5b00346. 25912298

[10] Lu Y, Liu X, Jiao Y, Xiong X, Wang E, Wang X, et al. Periostin promotes liver steatosis and hypertriglyceridemia through downregulation of PPARα. J Clin Invest 2014; 124: 3501–13. doi: 10.1172/JCI74438. 25003192 PMC4109546

[11] Zhong H, Chen K, Feng M, Shao W, Wu J, Chen K Genipin alleviates high-fat diet-induced hyperlipidemia and hepatic lipid accumulation in mice via miR-142a-5p/SREBP-1c axis. FEBS J 2018; 285: 501–17. doi: 10.1111/febs.14349. 29197188

[12] Wu BN, Kuo KK, Chen YH, Chang CT, Huang HT, Chai CY, et al. Theophylline-based KMUP-1 improves steatohepatitis via MMP-9/IL-10 and lipolysis via HSL/p-HSL in obese mice. Int J Mol Sci 2016; 17: 1345. doi: 10.3390/ijms17081345. PMC5000741 27548140

[13] Woo M, Song YO, Kang KH, Noh JS Anti-obesity effects of collagen peptide derived from skate (Raja kenojei) skin through regulation of lipid metabolism. Mar Drugs 2018; 16: 306. doi: 10.3390/md16090306. PMC6164805 30200239

[14] Lee YH, Jin B, Lee SH, Song M, Bae H, Min BJ, et al. Herbal formula HT048 attenuates diet-induced obesity by improving hepatic lipid metabolism and insulin resistance in obese rats. Molecules 2016; 21: 1424. doi: 10.3390/molecules21111424. PMC6274173 27792149

[15] Ahn H, Go GW Pinus densiflora bark extract (PineXol) decreases adiposity in mice by down-regulation of hepatic de novo lipogenesis and adipogenesis in white adipose tissue. J Microbiol Biotechnol 2017; 27: 660–7. doi: 10.4014/jmb.1612.12037. 28081360

[16] Mahendra Kumar C, Singh SA Bioactive lignans from sesame (Sesamum indicum L.): evaluation of their antioxidant and antibacterial effects for food applications. J Food Sci Technol 2015; 52: 2934–41. doi: 10.1007/s13197-014-1334-6. 25892793 PMC4397349

[17] Ying Z, Kherada N, Kampfrath T, Mihai G, Simonetti O, Desikan R, et al. A modified sesamol derivative inhibits progression of atherosclerosis. Arterioscler Thromb Vasc Biol 2011; 31: 536–42. doi: 10.1161/ATVBAHA.110.219287. 21183734 PMC5343762

[18] Shimizu S, Fujii G, Takahashi M, Nakanishi R, Komiya M, Shimura M, et al. Sesamol suppresses cyclooxygenase-2 transcriptional activity in colon cancer cells and modifies intestinal polyp development in Apc (Min/+) mice. J Clin Biochem Nutr 2014; 54: 95–101. doi: 10.3164/jcbn.13-91. 24688218 PMC3947973

[19] Hemalatha G, Pugalendi KV, Saravanan R Modulatory effect of sesamol on DOCA-salt-induced oxidative stress in uninephrectomized hypertensive rats. Mol Cell Biochem 2013; 379: 255–65. doi: 10.1007/s11010-013-1647-1. 23576423 PMC3666123

[20] Chennuru A, Saleem MT Antioxidant, lipid lowering, and membrane stabilization effect of sesamol against doxorubicin-induced cardiomyopathy in experimental rats. Biomed Res Int 2013; 2013: 934239. doi: 10.1155/2013/934239. 24228260 PMC3818820

[21] Go G, Sung JS, Jee SC, Kim M, Jang WH, Kang KY, et al. In vitro anti-obesity effects of sesamol mediated by adenosine monophosphate-activated protein kinase and mitogen-activated protein kinase signaling in 3T3-L1 cells. Food Sci Biotechnol 2017; 26: 195–200. doi: 10.1007/s10068-017-0026-1 30263528 PMC6049478

[22] Liu Z, Qiao Q, Sun Y, Chen Y, Ren B, Liu X Sesamol ameliorates diet-induced obesity in C57BL/6J mice and suppresses adipogenesis in 3T3-L1 cells via regulating mitochondria-lipid metabolism. Mol Nutr Food Res 2017; 61: 1600717. doi: 10.1002/mnfr.201600717. 28012248

[23] Pan MH, Chen JW, Kong ZL, Wu JC, Ho CT, Lai CS Attenuation by tetrahydrocurcumin of adiposity and hepatic steatosis in mice with high-fat-diet-induced obesity. J Agric Food Chem 2018; 66: 12685–95. doi: 10.1021/acs.jafc.8b04624. 30415544

[24] Ding Y, Gu Z, Wang Y, Wang S, Chen H, Zhang H, et al. Clove extract functions as a natural fatty acid synthesis inhibitor and prevents obesity in a mouse model. Food Funct 2017; 8: 2847–56. doi: 10.1039/c7fo00096k. 28726934

[25] Ma S, Jing F, Xu C, Zhou L, Song Y, Yu C, et al. Thyrotropin and obesity: increased adipose triglyceride content through glycerol-3-phosphate acyltransferase 3. Sci Rep 2015; 5: 7633. doi: 10.1038/srep07633. 25559747 PMC4284501

[26] Jiang C, Xie C, Lv Y, Li J, Krausz KW, Shi J, et al. Intestine-selective farnesoid X receptor inhibition improves obesity-related metabolic dysfunction. Nat Commun 2015; 6: 10166. doi: 10.1038/ncomms10166. 26670557 PMC4682112

[27] Ipsen DH, Lykkesfeldt J, Tveden-Nyborg P Molecular mechanisms of hepatic lipid accumulation in non-alcoholic fatty liver disease. Cell Mol Life Sci 2018; 75: 3313–27. doi: 10.1007/s00018-018-2860-6. 29936596 PMC6105174

[28] Kwon EY, Jung UJ, Park T, Yun JW, Choi MS Luteolin attenuates hepatic steatosis and insulin resistance through the interplay between the liver and adipose tissue in mice with diet-induced obesity. Diabetes 2015; 64: 1658–69. doi: 10.2337/db14-0631. 25524918

[29] Mo L, Shen J, Liu Q, Zhang Y, Kuang J, Pu S, et al. Irisin is regulated by CAR in liver and is a mediator of hepatic glucose and lipid metabolism. Mol Endocrinol 2016; 30: 533–42. doi: 10.1210/me.2015-1292. 27007446 PMC5414639

[30] Li Y, Zalzala M, Jadhav K, Xu Y, Kasumov T, Yin L, et al. Carboxylesterase 2 prevents liver steatosis by modulating lipolysis, endoplasmic reticulum stress, and lipogenesis and is regulated by hepatocyte nuclear factor 4 alpha in mice. Hepatology 2016; 63: 1860–74. doi: 10.1002/hep.28472. 26806650 PMC4874867

[31] Yang W, Huang L, Gao J, Wen S, Tai Y, Chen M, et al. Betaine attenuates chronic alcoholinduced fatty liver by broadly regulating hepatic lipid metabolism. Mol Med Rep 2017; 16: 5225–34. doi: 10.3892/mmr.2017.7295. 28849079 PMC5647077

[32] Softic S, Cohen DE, Kahn CR Role of dietary fructose and hepatic De Novo lipogenesis in fatty liver disease. Dig Dis Sci 2016; 61: 1282–93. doi: 10.1007/s10620-016-4054-0. 26856717 PMC4838515

[33] Softic S, Gupta MK, Wang GX, Fujisaka S, O’Neill BT, Rao TN, et al. Divergent effects of glucose and fructose on hepatic lipogenesis and insulin signaling. J Clin Invest 2017; 127: 4059–74. doi: 10.1172/JCI94585. 28972537 PMC5663363

[34] Andrade JM, Paraiso AF, de Oliveira MV, Martins AM, Neto JF, Guimaraes AL, et al. Resveratrol attenuates hepatic steatosis in high-fat fed mice by decreasing lipogenesis and inflammation. Nutrition 2014; 30: 915–19. doi: 10.1016/j.nut.2013.11.016. 24985011

[35] Bolsoni-Lopes A, Alonso-Vale MI Lipolysis and lipases in white adipose tissue – An update. Arch Endocrinol Metab 2015; 59: 335–42. doi: 10.1590/2359-3997000000067 26331321

[36] Watt MJ, Holmes AG, Pinnamaneni SK, Garnham AP, Steinberg GR, Kemp BE, et al. Regulation of HSL serine phosphorylation in skeletal muscle and adipose tissue. Am J Physiol Endocrinol Metab 2006; 290: E500–8. doi: 10.1152/ajpendo.00361.2005. 16188906

[37] Hsiao PJ, Chiou HC, Jiang HJ, Lee MY, Hsieh TJ, Kuo KK Pioglitazone enhances cytosolic lipolysis, beta-oxidation and autophagy to ameliorate hepatic steatosis. Sci Rep 2017; 7: 9030. doi: 10.1038/s41598-017-09702-3. 28831172 PMC5567271

[38] Koutsari C, Jensen MD Thematic review series: patient-oriented research. Free fatty acid metabolism in human obesity. J Lipid Res 2006; 47: 1643–50. doi: 10.1194/jlr.R600011-JLR200. 16685078

[39] Zhang H, Dellsperger KC, Zhang C The link between metabolic abnormalities and endothelial dysfunction in type 2 diabetes: an update. Basic Res Cardiol 2012; 107: 237. doi: 10.1007/s00395-011-0237-1. 22189563 PMC5512574

[40] Lewis GF, Carpentier A, Adeli K, Giacca A Disordered fat storage and mobilization in the pathogenesis of insulin resistance and type 2 diabetes. Endocr Rev 2002; 23: 201–29. doi: 10.1210/edrv.23.2.0461. 11943743

[41] Serra D, Mera P, Malandrino MI, Mir JF, Herrero L Mitochondrial fatty acid oxidation in obesity. Antioxid Redox Signal 2013; 19: 269–84. doi: 10.1089/ars.2012.4875. 22900819 PMC3691913

[42] He F, Jin JQ, Qin QQ, Zheng YQ, Li TT, Zhang Y, et al. Resistin regulates fatty acid beta oxidation by suppressing expression of peroxisome proliferator activator receptor gamma-coactivator 1alpha (PGC-1α). Cell Physiol Biochem 2018; 46: 2165–72. doi: 10.1159/000489546. 29730652

[43] Chen SD, Yang DI, Lin TK, Shaw FZ, Liou CW, Chuang YC Roles of oxidative stress, apoptosis, PGC-1α and mitochondrial biogenesis in cerebral ischemia. Int J Mol Sci 2011; 12: 7199–215. doi: 10.3390/ijms12107199. 22072942 PMC3211033

[44] Henique C, Mansouri A, Fumey G, Lenoir V, Girard J, Bouillaud F, et al. Increased mitochondrial fatty acid oxidation is sufficient to protect skeletal muscle cells from palmitate-induced apoptosis. J Biol Chem 2010; 285: 36818–27. doi: 10.1074/jbc.M110.170431. 20837491 PMC2978610

[45] Dai J, Liang K, Zhao S, Jia W, Liu Y, Wu H, et al. Chemoproteomics reveals baicalin activates hepatic CPT1 to ameliorate diet-induced obesity and hepatic steatosis. Proc Natl Acad Sci U S A 2018; 115: E5896–905. doi: 10.1073/pnas.1801745115. 29891721 PMC6042128

[46] Perfield JW, Ortinau LC, Pickering RT, Ruebel ML, Meers GM, Rector RS Altered hepatic lipid metabolism contributes to nonalcoholic fatty liver disease in leptin-deficient Ob/Ob mice. J Obes 2013; 2013: 296537. doi: 10.1155/2013/296537. 23401753 PMC3562693

[47] Herrema H, Zhou Y, Zhang D, Lee J, Salazar Hernandez MA, Shulman GI, et al. XBP1s is an anti-lipogenic protein. J Biol Chem 2016; 291: 17394–404. doi: 10.1074/jbc.M116.728949. 27325692 PMC5016136

[48] Laiglesia LM, Lorente-Cebrián S, Martínez-Fernández L, Sáinz N, Prieto-Hontoria PL, Burrell MA, et al. Maresin 1 mitigates liver steatosis in ob/ob and diet-induced obese mice. Int J Obes (Lond) 2018; 42: 572–79. doi: 10.1038/ijo.2017.226. 28895586

[49] Wang CC, Yen JH, Cheng YC, Lin CY, Hsieh CT, Gau RJ, et al. Polygala tenuifolia extract inhibits lipid accumulation in 3T3-L1 adipocytes and high-fat diet-induced obese mouse model and affects hepatic transcriptome and gut microbiota profiles. Food Nutr Res 2017; 61: 1379861. doi: 10.1080/16546628.2017.1379861. 29056891 PMC5642193

